# “*But* *how* *true* *that* *is*, *I* *do* *not* *know*”: the influence of written sources on the medicinal use of fungi across the western borderlands of the former Soviet Union

**DOI:** 10.1186/s43008-024-00156-7

**Published:** 2024-08-05

**Authors:** Julia Prakofjewa, Matteo Sartori, Raivo Kalle, Łukasz Łuczaj, Małgorzata Karbarz, Giulia Mattalia, Povilas Šarka, Baiba Prūse, Nataliya Stryamets, Martin Anegg, Natalia Kuznetsova, Valeria Kolosova, Olga Belichenko, Muhammad Abdul Aziz, Andrea Pieroni, Renata Sõukand

**Affiliations:** 1 Department of Environmental Sciences, Informatics and Statistics, Ca’ Foscari University of Venice, Venice, Italy; 2 Estonian Literary Museum, Tartu, Estonia; 3 University of Gastronomic Sciences, Pollenzo, Italy; 4 Institute of Biology, University of Rzeszów, Rzeszów, Poland; 5 Institut de Ciència i Tecnologia Ambientals, Universitat Autònoma de Barcelona (ICTA-UAB), Barcelona, Spain; 6 Botanical Garden of Vilnius University, Vilnius, Lithuania; 7 Athena Institute, Vrije Universiteit Amsterdam, Amsterdam, The Netherlands; 8 Roztochya Nature Reserve, Ivano-Frankove, Ukraine; 9 Faculty of Forest Sciences, School of Forest Management, Swedish University of Agricultural Sciences, Skinnskatteberg, Sweden; 10 Utz Group, Bremgarten, Switzerland; 11 Università Cattolica del Sacro Cuore, Milan, Italy; 12 Muséum National d’Histoire Naturelle, Paris, France; 13 Medical Analysis Department, Tishk International University, Erbil, Iraq

**Keywords:** Fungi, Eastern Europe, Medicinal fungi, Lichen, Historical ethnomycology, Knowledge circulation, Herbals, Local ecological knowledge, Ethnomycology, Book knowledge

## Abstract

Fungi have been used for medicinal purposes for many centuries. This study, based on 35 historical written sources and 581 in-depth semi-structured interviews from eight countries in the western borderlands of the former Soviet Union, investigates the medicinal use of fungi by local communities. We compared the taxa and uses obtained from fieldwork and historical sources with works that advocated fungi use within Soviet herbals, representing the centralised medical system. During fieldwork, we identified eight locally used fungi and one lichen. The highest numbers of medicinal uses were documented in Russia, Estonia and Ukraine. Studies published before the Soviet era listed 21 fungal taxa and one lichen species used in the study region. However, only six of these taxa were mentioned as used by people in our field studies (*Amanita* *muscaria*, *Boletus* *edulis*, *Lycoperdon*, *Morchella*, *Phallus* *impudicus* and *Cetraria* *islandica*). Notably, these same six taxa were consistently endorsed in Soviet herbals. Of the remaining three taxa recorded in the fieldwork, none were mentioned in historical written sources. However, they were promoted either in Soviet herbals (*Inonotus* *obliquus*, *Kombucha*) or later popular publications (*Cantharellus* *cibarius*). This highlights the significant influence of written sources on the use of fungi for medicinal purposes within the studied local communities.

**Supplementary Information:**

The online version contains supplementary material available at 10.1186/s43008-024-00156-7.

## INTRODUCTION

The importance of the links between humans and fungi is increasingly gaining recognition within the scientific literature (Yamin-Pasternak 2011; Pérez-Moreno et al. 2020a, b; Haro-Luna et al. 2022; Stryamets et al. 2022; Gorriz-Mifsud et al. 2023), which encompasses economic contributions, medicinal applications, nutritional value, ecological impact (Kovalčík 2014; de Aragón et al. 2011) and cultural traditions (Tsing 2015). Ethnomycology, the study of this interplay, has seen a surge in interest, with fungal knowledge being analysed across different ethnic groups and time periods (Wasson and Wasson 1957; Egli et al. 2006; Boa 2004). Significant contributions to this field have come from diverse geographical regions, including notable research in Asia (Upadhya and Navi 2023), America (Ríos-García et al. 2023) and Africa (Osemwegie et al. 2014), which underscore the rich traditional knowledge and use value of fungi in these areas. However, the focus on European ethnomycological studies appears less extensive (Comandini and Rinaldi 2020; Gründemann et al. 2020), with notable research concentrated in specific countries, such as Finland (Härkönen 1998; Turtiainen et al. 2012), Estonia (Jürgenson 2022), Poland (Marczyk 2003; Kotowski 2019; Kotowski et al. 2019; Łuczaj and Nieroda 2011; Łuczaj and Köhler 2014; Trojanowska 2001), Scotland (Dyke and Newton 1999), Spain (De Román and Boa 2004; Palahí et al. 2009), Italy (Pieroni 2016), Lithuania (Radušienė and Janulis 2004; Džekčioriūtė-Medeišienė 2016), Latvia (Donis and Strauppe 2011; Lībiete 2017), Romania (Łuczaj et al. 2015; Papp et al. 2017), Hungary (Győző, 2019), the European part of Russia (Azeem et al. 2020; Belichenko 2022), Belarus (Kotowski et al. 2019) and Ukraine (Sõukand and Pieroni 2016; Stryamets et al. 2022). These studies offer valuable insights into local ethnomycological knowledge and practices, and the importance of fungi among various ethnic groups. Nevertheless, the research has not extended to communities in borderlands, which have been divided by historical events. The twentieth century witnessed significant shifts that profoundly altered Europe’s geopolitical landscape and local ecological knowledge, impacting the understanding and use of natural resources, including fungi, across various regions (Rosa-Gruszecka et al. 2017).

Europe’s biological diversity fosters a rich cultural mosaic with various attitudes towards fungi, ranging from mycophilic (fungus-loving) to mycophobic (fungus-fearing) (Peintner et al. 2013; Comandini et al. 2018). In regions with mycophilic traditions, such as Northern, Central and Eastern Europe, as well as parts of the Mediterranean, there is a longstanding tradition of foraging for wild fungi, incorporating them into traditional culinary practices and utilising them for medicinal purposes (Hobbs 2002; Dugan 2008; Grienke et al. 2014). In these regions, fungi are often celebrated and deeply ingrained in the cultural context, with their use being passed down through generations. This is contrasted by more mycophobic attitudes in other parts of Europe, where fungi are often viewed with suspicion and caution, likely a legacy of historical events or the lack of traditional mycological knowledge.

The historical origins of using fungi for medicinal purposes extend far back in time (Emmons 1961). One remarkable example can be traced to Dioscorides, a Greek physician from the first century, who diligently recorded the therapeutic benefits of fungi. Despite his association of fungi with plants and lingering uncertainties about their origin, Dioscorides (2005) recognised and documented their healing properties, contributing to the rich heritage of fungi in medicine. Later, commenting on Dioscorides’ text, the Italian naturalist Pietro Andrea Mattioli (1565) pondered the possible medicinal uses of fungi while reflecting on their potential toxicity.

The expanding body of scientific literature emphasises the significant influence fungi exert on human well-being, highlighting the need for continued investigation and understanding of these essential interrelations in the contemporary world (Gafforov et al. 2023). Since the middle of the nineteenth century, there has been an increasing fascination with the potential medicinal application of fungi across Northern, Central and Eastern Europe, as noted by various scholars (Redwood 1857; Mamedov et al. 2005; Svanberg 2018; Comandini and Rinaldi 2020). For instance, in 1864, German pharmacologist Dragendorff conducted research on the medicinal properties of a fungus found on birch trees, which he described as “similar to Fomes fomentarius” (Dragendorff 1864: 5). This historical context underscores not only the persistent curiosity about the medicinal value of fungi in the region, but also early research endeavours, the importance of accurate identification and preparation, and the commitment to mitigating health risks associated with fungi (Spuhl-Rotalia 1897; Leppik 1936). Moreover, the establishment of the International Journal of Medicinal Mushrooms in 1999 marked a significant milestone. This journal has served as a springboard for disseminating knowledge and research findings, specifically fostering a global scientific dialogue on the topic. The contribution of such scholars as Wasser and Weis (1999) and Stamets (2011) have been instrumental in the recent fungal renaissance. As large and small pharmaceutical companies begin to explore the bioactive compounds found in fungi, the potential for new drug development is vast. Health care professionals are increasingly incorporating mushroom-based supplements into their practices, acknowledging their role in promoting health and potentially aiding in the treatment of diseases (Gupta et al. 2020). Nevertheless, despite this keen interest, the exploration of medicinal fungi in European countries has been relatively confined to certain areas or specific fungal species, such as *Inonotus* *obliquus* (Shashkina et al. 2006; Szychowski et al. 2021) and *Amanita* *muscaria* (Biziulevičius and Vaitkuviene 2007; Feeney 2013).

The circulation of knowledge about medicinal fungi, as with any kind of knowledge, is a complex process that involves changes and adaptations over time (Jacob 2017). It is often shared through oral traditions and practical experiences (Comandini and Rinaldi 2020). However, the impact of written sources in shaping such knowledge remains an unexplored area (Turner and Cuerrier 2022). Therefore, this study aims to examine the role of written sources with regard to the use of fungi in medicine, taking as an example the ethnic groups residing in the western borderlands of the former Soviet Union. The research encompasses three main aspects: (1) documenting the local ecological knowledge surrounding the medicinal use of fungi in the studied areas, which include border regions between Finland and Russia; Estonia and Russia; Poland, Lithuania and Belarus; and Ukraine and Romania; (2) conducting a cross-cultural comparison of their medicinal uses; and (3) assessing temporal changes in their use as well as knowledge circulation. This research is crucial considering the evolving ways in which knowledge is shared and disseminated nowadays.

## METHODS

### Study area

**Fig. 1:**
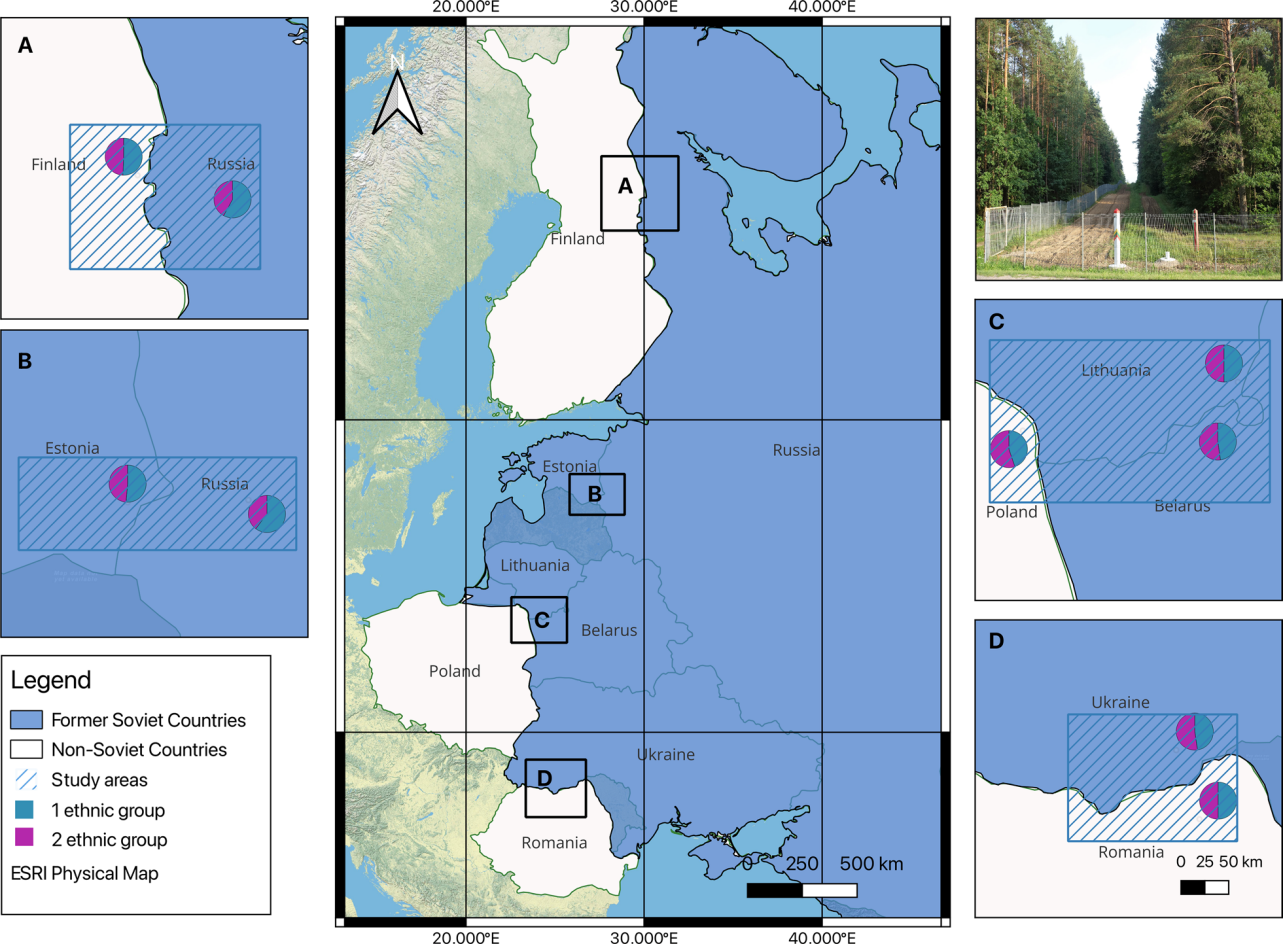
Specific study areas selected: **A** Karelia (Finnish-Russian Borderland); **B** Setomaa (Estonian-Russian Borderland); **C** Polish-Lithuanian-Belarusian Borderland; **D** Bukovina (Ukrainian-Romanian Borderland). Designed by JP in QGIS 3.30.3

Our study region includes the frontier areas between Finland and Russia; Estonia and Russia; Poland, Lithuania and Belarus; and Ukraine and Romania (Fig. 1).

The study area is located along the former border axis, covering Finland and territories that were formerly under the centralised administration of the Soviet Union, including Russia, Estonia, Lithuania, Belarus and Ukraine, as well as post-socialist Poland and Romania.

The study region spans Northern, Central and Eastern Europe, encompassing diverse landscapes and climates. This geographical area is characterised by dynamic vegetation patterns and constantly shifting climatic conditions, which significantly contribute to the richness and variety of fungal species. Moreover, the study borderland displays notable heterogeneity, with pronounced environmental variations between its northern and southern limits. These variations exert considerable influence on forest cover and composition (Palang et al. 1998; Solon 2011). Soviet forest management led to the extensive replacement of natural forest communities with coniferous forests (Kuemmerle et al. 2006). However, following the collapse of the Soviet Union, many abandoned agricultural fields have gradually transformed into forests once again, a process that has continued steadily over time (Kuemmerle et al. 2011; Prishchepov et al. 2012).

### Data collection and analysis

Field data were collected in the summers of 2018 and 2019 via 581 in-depth semi-structured interviews at 18 research sites in eight countries among nine ethnic and/or linguistic groups divided by state borders. Specifically, we carried out interviews with 34 Finns and 33 Karelians in Finland. In Russia, we interviewed 29 Karelians and 21 Russians living in the Republic of Karelia. We also conducted interviews with 37 Setos and 35 Võros in Estonia. On the Russian side of the border, we included data from 25 Setos and 38 Russians living in the Pskov region. We also recorded interviews among 33 Lithuanians and 40 Poles in Belarus. The survey in Lithuania was completed via interviews with 30 Lithuanians and 37 Poles. In addition, we selected 32 Lithuanians and 32 Poles to interview in Poland. In Ukraine, interviews were conducted with 31 Hutsuls and 34 Romanians. Finally, data was gathered in Romania via interviews with 30 Hutsuls and 30 Romanians.

Generally, the inhabitants of the studied territories are proficient in two or more languages. Russian serves as a common language across these regions, with the notable exceptions of Finland and Romania. The majority of those interviewed are retirees who previously worked on collective farms, particularly in Belarus, Lithuania, Estonia, Russia and Ukraine. Each of the interviewees has resided in a rural location for at least the last 15 years. The selected rural areas are predominantly near forests, facilitating direct observation of ongoing practical activities.

The interviewees were conveniently selected among local inhabitants willing to share their LEK. The interviews ranged from approximately one to three hours, and discussions covered various topics, including the uses of wild food plants and ethnomedicine in general. Prior informed consent was obtained, and the Code of Ethics of the International Society of Ethnobiology (ISE 2006) was strictly followed. The study received approval from the Ethical Committee of Ca’ Foscari University of Venice. The uses of fungi, if any, were named along with the use of plants, as only a limited number of people mentioned using fungi for medicinal purposes. Interview questions covering all disease categories addressed the use of fungi to treat various ailments. The interviews were conducted in the local language(s) according to the interviewee’s preference. Interviews were generally held in Finnish in Finland, in Russian in Russia; in Estonian in Estonia; in Lithuanian, Belarusian, Polish and Russian in Lithuania; in Lithuanian and Polish in Poland; in Lithuanian, Polish, Belarusian and Russian in Belarus; in Ukrainian, Romanian and Russian in Ukraine; and in Romanian and Ukrainian in Romania.

Fresh or dried fungal voucher specimens were collected where possible and stored in the herbarium of Ca’ Foscari University of Venice (UVV) or in each of the non-EU countries.

The interviews were transcribed, and the data was structured in an Excel file according to Use Reports (UR), which included the interview code, the fungus’s scientific name and family, medicinal use and time period (Appendix A1). Period of use was categorised into current (still in use) and past (abandoned either during the childhood or adulthood of the interviewee), the latter usually referring to periods before the 1990s. Fungal nomenclature followed the Index Fungorum (2023). *Lycoperdon* and *Morchella* were identified at the genus level since interviewees often did not distinguish between various species regarding medicinal use. The identification of *Kombucha* (“tea fungus”) was retained as a folk taxon because our interviewees perceived this symbiotic culture as a type of fungus. For the analysis of documented narratives, we employed grounded theory (Pidgeon and Henwood 2004) and content analysis techniques (Krippendorff 2018).

The information gathered during the interviews was used for sentiment analysis (Feldman 2013). This analysis aimed to assess the circulation of knowledge and perception of medicinal fungi use. We performed this task manually, enabling the categorisation of participants’ perceptions as positive, negative, neutral or not stated. We identified unsuccessful and negative usage experiences within the context of negative sentiment, while in the positive sentiment category, we acknowledge positive and healing experiences. Meanwhile, the neutral category encompasses valuable statements of fact.

To connect current fieldwork with historical knowledge, data on the use of medicinal fungi in the studied regions from pre-Soviet sources was added. This included selecting key texts in Polish, German, Lithuanian and Russian to obtain a broad overview. The ethnobotanical historical works in the German language were extracted from the publications authored by Anegg et al. in (2021) and (2022). We reviewed a total of 20 works that were published between the late eighteenth century and the beginning of the twentieth century (Jundziłł 1791; Friebe 1805; von Luce 1829; Tyszkiewicz 1847; Kirkor 1858; Hoelzl 1861; Bobrovskiy 1863; Wiedemann 1876; Cholovskiy 1882; Rostafiński 1883; Orzeszkowa 1891; Alksnis 1894; Nikiforovskiy 1895; Kolbuszowski 1896; Wereńko 1896; Federowski 1897; Bermann and Ludwig 1904; Ludwig 1905; Petkevič 1911; Muszyński 1927). The intended audience for all these publications was the scholarly community. The content of these works, while sometimes incorporating local knowledge about fungi, was primarily scientific. They included descriptions of medicinal properties, but the approach was more taxonomic and botanical rather than focusing on practical, everyday use by the general population. Nevertheless, despite the lack of reported medicinal uses of fungi in written sources from the pre-Soviet period concerning the Karelian region (Kolosova et al. 2022), these works serve as an invaluable resource for comprehending the historically recorded applications of medicinal plants and fungi in the specified regions. This inclusion facilitates a comparative study of the traditional ecological knowledge documented in the sources pre-dating the Soviet era.

During the Soviet period, the scope expanded beyond the scholarly community to the general public. Publications were aimed not only at academics but also at educating and guiding local people in identifying, collecting and utilising medicinal fungi. Moreover, during the Soviet era, the focus shifted towards making scientific knowledge, including herbal medicine, accessible to all citizens. This shift led to herbals being written in simpler language with practical, user-friendly guidance, reflecting broader trends in literacy, education and public access to scientific knowledge (Bexultanova et al. 2022). The use of Russian as a lingua franca significantly expanded the accessibility and readership of these books across diverse ethnic groups. If pre-Soviet works served as a foundation for scientific understanding and classification, Soviet publications aimed to democratise this knowledge, making it accessible to and usable by the wider population.

We selected 15 books published in the Soviet period. In total, the dataset encompassed 35 published works. Furthermore, we gathered information from a selection of 15 primary official (censored) Soviet herbals, books that featured the medicinal use of fungi and had a circulation exceeding 10,000 copies (Zemlinskiy 1958; Popov 1968; Kondratenko et al. 1965; Gammerman 1967; Sklyarevskiy and Gubanov 1970; Turova 1974; Yurkevich et al. 1976; Stekol’nikov and Murokh 1979; Zhurba 1988; Šmiarko 1989; Shamruk 1990; Popov et al. 1990; Pastushenkov et al. 1990; Grinkevich et al. 1991; and Zadorozhnyy et al. 1988).

**Fig. 2:**
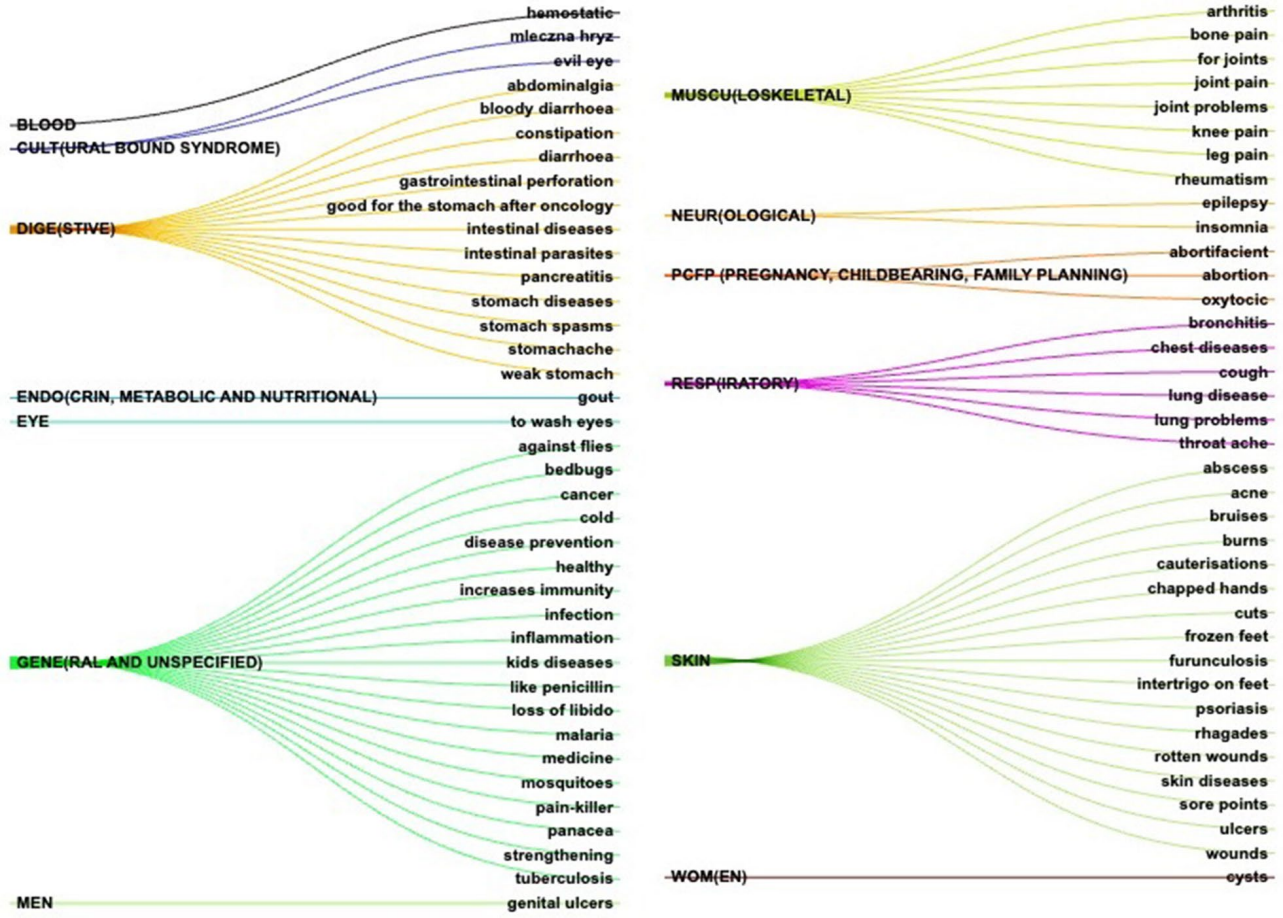
Correspondence between medicinal disease categories and emic diseases that are treated with fungi and lichens

Emic disease names were correlated with the medicinal categories of the International Classification of Primary Care, 2nd edition (ICPC-2, Updated March 2003) (hereafter referred to as etic disease categories). This correlation facilitated comparisons with pre-Soviet and Soviet-published works (Fig. 2). In some instances, assigning a specific disease to a particular category might involve subjectivity, such as in cases where designations of chest ailments or bronchitis recorded between the end of the eighteenth and the beginning of the twentieth century could potentially align with the general or respiratory disease categories. Furthermore, the analysis included the culture-bound disease category for culturally specific health conditions. This correlation provides a deeper understanding of the connections between local and traditional disease names and their corresponding classifications within the ICPC-2 system, thereby enabling more extensive research and analysis across various historical sources.

We divided the obtained data into three different stages of knowledge circulation (see Prakofjewa et al., 2024):

1. Eighteenth-twentieth centuries—the data reported in pre-Soviet and Soviet written sources;
2. ~ 1990s—The uses reported by our interviewees as once utilised but now abandoned (this date largely corresponds to the decade of the collapse of the Soviet Union, as the majority of uses were discontinued at that time according to our interviewees);
3. 2010s—The uses reported as currently in use during our fieldwork in 2018–2019.

### DNA barcoding

Fungal voucher specimens were identified first morphologically and then with DNA barcoding (Schindel and Miller 2005; Schoch et al. 2012), following the guidelines of widely accepted methods for DNA barcoding of fungi (Eberhardt 2012). The DNA from fungi was extracted from a small part of the sporocarp (ca. 1 mm^3^ of dry mycelium taken from the fruiting body) using a Plant and Fungi DNA Purification Kit (Eurx), following standard protocol. The PCR cocktail consisted of a 4 μl DNA extract, 0.5 μl of each of the primers (ITS5/ITS1f and ITS4 in 10 nmol concentration) and a 5 μl Type-it Microsatellite PCR Kit (Qiagen). PCR was carried out using the following thermocycling conditions: an initial 15 min at 95 °C, followed by 35 cycles at 95 °C for 30 s, 55 °C for 30 s, 72 °C for 1 min and a final cycle of 10 min at 72 °C. The PCR products were estimated by running a 5 μl DNA amplicon on 1.5% agarose gel for 30 min. The PCR products were sequenced using ITS4 or ITS5 primers at the Molecular Biology Techniques Laboratory at Adam Mickiewicz University (Poznań, Poland). Using the BLAST tool, we compared the obtained sequences with published sequences in the BOLDSYSTEMS databases. A positive identification of a specimen was confirmed if it shared > 97% ITS region sequence identity with the reference sequence. Nuclear ITS sequences obtained in this study have been deposited in GenBank (with the accession numbers listed in Table 1).

**Table 1:** Molecular identification of fungal samples analysed in this study and GenBank accession numbers for ITS sequences

Morphological identification	Herbarium numbers	Molecular identification	Similarity (%)	Best match sequence	Accession number
*Amanita* *muscaria*	EEFU27	*Amanita* *muscaria*	99.76	EU071906	OR668594
*Amanita* *muscaria*	EEFU28	*Amanita* *muscaria*	100	AB081295	OR668595
*Amanita* *muscaria*	PSLT05	*Amanita* *muscaria*	100	AB081295	OR668596
*Boletus* *edulis*	KARSE07a	*Boletus* *edulis*	99.33	KC750229	OR668597
*Boletus* *edulis*	ROFU04	*Boletus* *edulis*	99.84	JN029381	OR668598
*Boletus* *edulis*	EEFU20	*Boletus* *edulis*	99.67	KC750229	OR668599
*Boletus* *edulis*	EEFU12	*Boletus* *edulis*	98.84	JN029381	OR668600
*Cantharellus* *cibarius*	KARSE04	*Cantharellus* *cibarius*	99.34	JN944019	OR668601
*Cantharellus* *cibarius*	PSLT25	*Cantharellus* *cibarius*	99.17	JN944019	OR668602
*Inonotus* *obliquus*	KARSE19	*Inonotus* *obliquus*	98.99	OP942274.1	PP461800
*Inonotus* *obliquus*	KARSE16	*Inonotus* *obliquus*	98.42	PP346419.1	PP461803
*Lycoperdon* sp.	EEFU25	*Lycoperdon* *sp.*	99.35	KT875054	OR668603
*Morchella* spp.	ROFU01	*Morchella* *sp.*	99.64	JQ723060	OR668604

## RESULTS

### Current use of medicinal fungi and lichens in the western borderlands of the former Soviet Union

**Fig. 3:**
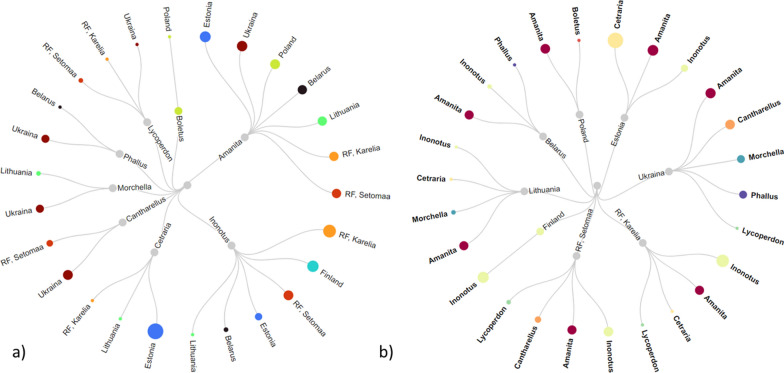
Distribution of the use of fungi and lichens by taxon **a** and country **b**

Eight fungal and one lichen taxa were used or remembered as having been utilised in 161 Use Reports (UR) (Table 2). The highest number of UR was recorded for *Amanita* *muscaria* (66 UR) and *Inonotus* *obliquus* (44 UR, of those 24 from Russia). *Amanita* *muscaria* was used in six countries (seven regions), while *Inonotus* *obliquus* was utilised in five countries (six regions) (Fig. 3a). The least reported taxa were *Lycoperdon* and *Kombucha* (4 UR). The highest number of taxa (five) was reported in Ukraine, followed by Lithuania and both regions of Russia, with four taxa reported in each (Fig. 3b). There were no reports from Romania, one from Finland and two from Poland.

**Table 2:** Fungi and lichen uses reported by our interviewees with the number of UR summarised over field study sites

Taxon	Local name (country)	Part used	Preparation	Treated illness (UR if more than one)
*Amanita* *muscaria*	*Muchamory* *(krasnyje)* (BY); *muchamor* (LT, PL); *muchumor* (PL); *musiomirai* (LT); *musmirė* (LT); *mukhomor* *(*RU); *muchomor* (PL); *mukhomor* (UA); *hadiar* (UA); *chervonyi* *mukhomor* (UA); *kärbseseen* (EE)	Fungus	Infused in alcohol for topic application	Joint pain (17); leg pain (2); rheumatism; bruise; frozen foot; acne
			Infused in alcohol for drinking in small amounts	Throat ache, flu; good for health; inflammation; joint pain (3)
			Fermented, then added vodka; drunk	Climax; epilepsy; neurosis; cancer; schizophrenia; stress
			Fermented, then added vodka; topic application	Headache; bruise; cuts
			Fermented, then added vodka; rubbed in	Joint pain (2); muscle pain; pain-killer
			Left on the table	Disease prevention (to kill the flies) (26)
			Left on the table with milk	
			Left on the table with sugar	
			Boiled (roasted) with sugar	
			Boiled with milk	
			Dried, then burned as incense	Disease prevention (to repel mosquitos)
*Boletus* *edulis*	*Belyj* *grib* (RU); *borowik* (PL, RU)	Fungus	Not stated	Cancer
			Boiled, topic application	Skin diseases
*Cantharellus* *cibarius*	*Lisichka* (RU); *lysychka* (UA); *svichka* (UA)	Fungus	Dried and eaten	Like antibiotic; cancer
			Tincture	Cancer (2); immunity boosting
			Raw (dried and with water)	Parasites (worms) (6)
*Cetraria* *islandica*	*Põdrasamma* (EE); *islandi* *samblik* (EE); *tsetrariya* *islandskaya* (RU); *mokh* *islandskiy* (RU); *tsentarium* (RU); *elninės* *kerpės* (LT)	Sporophores	Tea	Bronchitis (2); cough (23); lung disease; medicine
*Inonotus* *obliquus*	*Chaga* (RU); *berezovyy* *grib* (RU); *pakuri* (FIN); *pakurikääpä* (FIN); *beržo* *grybas* (LT); *chaga* (LT)	Sclerotia	Infused in alcohol for drinking in small amounts	Stomachache; panacea; cancer (2); cysts
			Decoction for drinking	Stomach diseases (9); hangover (2); panacea (6); intestinal diseases; pancreatitis; cancer (8); psoriasis; cough; mastopathies; tonic (2); constipation; myoma; rejuvenating; cleaning; not stated
			Decoction for topic application	Wash eyes
			Dried	Healthy
			Fresh, soaked	
			Crumbled	
			Grinded	
			Not stated	Stomach and abdomen pain
*Lycoperdon* *sp.*	*Hryb* *porchaŭka* (BY); *dozhdevik*, *dedushkin* *tabak*, *dymyashchiysya* *grib* (RU); *porkhavka* (UA)	Spore	Dried, topic application	Cuts, wounds (2); burns; intertrigo on feet
*Morchella* sp.	*Smarčok* (PL, BY); *zbarciog* (RO)	Fungus	Infused in alcohol and drunk (in small amount)	Stomach pain (2); panacea (2)
			Infused in alcohol for topic application	Joint pain (4)
*Phallus* *impudicus*	*Vaniučy* *hryb* (BY); *viasiołka* (BY); *vesolka* (UA); *pucioasa* (RO)	Young fungus	Infused in (weak) alcohol, topic application	Panacea; rheumatic pain (4)
			Infused in (weak) alcohol, intake in small amount	Stomach pain
*Kombucha*	*Chaynyy* *grib* (RU)	“Fungus”	Fresh, topic application	Rheumatism, knees; cuts
		Fermented broth	Drunk	Good for stomach; sore throat

*BY* Belarus, *EE* Estonia, *FIN* Finland, *LT* Lithuania, *PL* Poland, *RO* Romania, *RU* Russia, *UA* Ukraine

Of the 161 UR, the highest numbers of uses were recorded in Russia (51), Estonia (33) and Ukraine (32), while the lowest number of use reports was from Belarus (11) and then Finland (12). The most treated disease categories were general (71 UR) and musculoskeletal (32 UR).

Of the 581 people interviewed, 110 (19%) used fungi for healing. Of them, 78 people (71%) named a single use and only 7 (6%) named four different uses. The largest numbers of medicinal fungi users were found in Estonia (30) and Russia (29), with *Inonotus* *obliquus* dominating the list of species. At the same time, all six interviewees using fungi in Finland admitted utilising the same taxon (*Inonotus* *obliquus*). Two-thirds of the UR were related to current use (103), while approximately one-third (58) were remembered from the past. There were no abandoned taxa among the recorded ones and no recently learned about taxa. Only a few taxa-use combinations had more than one person who mentioned their use. Regardless of the limited number of taxa and disease categories treated by the taxa, there were many modes of application and specific emic diseases or symptoms treated. The most widespread was the use of *Amanita* *muscaria* to alleviate joint and leg pain (in six regions by 19 people) and against flies (as a disease prevention tool) in diverse forms of preparation (in four regions by 26 people). The use of tea made with *Cetraria* *islandica* to treat cough (in three regions by 23 people) was especially prevalent among Setos in Estonia (named by 16 people). *Inonotus* *obliquus* was used against cancer in various forms of preparation (as a decoction or alcoholic extract) in six different regions; solely among Russians in the Republic of Karelia (RF) was it named by more than one person (4). In contrast, seven Karelians in RF reported using its decoction against stomach diseases.

### Sources and perception of ethnomycological knowledge

Fifty-five interviewees reported acquiring the studied knowledge from childhood, making it an integral part of their TEK, which interviewees considered quite evident. Adulthood family experience was another significant source of knowledge, with 41 interviewees stating that their understanding of medicinal use was grounded on experiences, both successful and unsuccessful, shared within their families. Notably, 24 interviewees mentioned encountering information about the use of fungi from sources beyond their immediate family, suggesting it was shared by individuals who were not relatives. However, one-third of our interviewees doubted the exact origin of the studied knowledge, emphasising that they had heard about it but lacked detailed information to confirm its trustworthiness as a source. Books and newspapers were cited as sources of ethnomycological knowledge by 12 interviewees, while only three mentioned acquiring information from the Internet. Eight interviewees highlighted that their knowledge originated from the Soviet procurement system in which mushrooms were purchased from the population.

Overall, we recorded a higher percentage (67%) of positive perceptions concerning the use of medicinal fungi in the study area. We observed that the most positive sentiments (45 mentions) involved the use of *Amanita* *muscaria*. The majority of interviewees held a firm belief in the positive effect of this fungus as a preventive measure against flies in the past, and this use is actually included in the name of the taxon in many languages of the studied regions. Acknowledging and exchanging knowledge about the toxic origins of the fungus, interviewees pointed out that *Amanita* *muscaria* is an effective remedy in medical applications. However, some doubts were also expressed about its usage (5 mentions).

Regarding *Inonotus* *obliquus*, 29 interviewees mentioned having a positive attitude towards its medicinal potential, while nine doubted its effectiveness. Similarly, 16 interviewees had a positive view on the use of *Cetraria* *islandica* in medicine, and seven expressed positive sentiments towards *Cantharellus* *cibarius*. Interestingly, negative connotations were also associated with *Cetraria* *islandica*, with five interviewees expressing concerns about its use. Additionally, *Inonotus* *obliquus* was mentioned negatively by three individuals.

### Historical use of medicinal fungi and lichens in the western borderlands of the former Soviet Union in written sources

Twenty-one fungal taxa (of which three were identified at the genus level) and one lichen species were reported in pre-Soviet written sources through 83 UR (Table 3; Appendix A2). The most popular taxa mentioned were *Amanita* *muscaria* (28 UR by 13 authors out of 20) and *Cetraria* *islandica* (14 UR by eight authors). *Amanita* *muscaria* was mainly reported to be used against general and musculoskeletal diseases, while *Cetraria* *islandica* was utilised to treat general and respiratory diseases. *Lycoperdon* was used in all four studied regions; *Amanita* *muscaria* and *Phallus* *impudicus* were reported for all regions apart from Western Ukraine. The most prevalent disease categories were skin (19 UR, eight taxa), general (18 UR, six taxa), digestive (15 UR, eight taxa) and musculoskeletal (11 UR, three taxa).

**Table 3:** The use of fungi for medicinal purposes documented in pre-Soviet written sources within the study area

Latin name and family (according to Index Fungorum)	The constituent name stated in the original	Local name	Medicinal use	Etic disease category	References
*Amanita* *muscaria* (Amanitaceae)	*Agaricus* *muscarius*; *amanita* *muscaria* L., Agaricaceae; *agaricus* *muscarius*, L.; A. Muscarius, L	*Masmires*, *mukhomor* *krasnyy*, *muchamor*, *muchomor*, *musmirė*, *mukhomor*, *muchomor* *czerwony*	“*Mleczna* *hryz*”	Culture-bound syndrome	Wereńko (1896)
			Arthritis	Musculoskeletal	Pietkiewicz, (1928); Wereńko (1896)
			Bed bugs	General and unspecified	Jundziłł, (1791)
			Bloody diarrhoea	Digestive	Kirkor (1858)
			Bone pain	Musculoskeletal	Alksnis (1894)
			Cauterisations	Skin	Alksnis (1894)
			Chest pain (bronchitis, pulmonary tuberculosis, emphysema, cardiac anomalies, probably also abdominalgia)	Respiratory	Alksnis (1894)
			Diarrhoea	Digestive	Tyszkiewicz (1847); Bobrovskiy (1863); Kirkor (1858); Cholovskiy (1882)
			Dysentery	Digestive	Tyszkiewicz (1847)
			Flies	General and unspecified	Jundziłł, (1791); Kolbuszowski (1896); Tyszkiewicz (1847); Bobrovskiy (1863); Nikiforovskiy, (1895)
			Joint pain	Musculoskeletal	Federowski (1897); Petkevič, (1911); Nikiforovskiy, (1895)
			Malaria	General and unspecified	Pietkiewicz (1928)
			Rhagades	Skin	Alksnis (1894)
			Rheumatism	Musculoskeletal	Muszyński (1927); Tyszkiewicz (1847); Wereńko (1896); Cholovskiy (1882)
			Sore points	Skin	Alksnis (1894)
			Stomach spasms	Digestive	Nikiforovskiy, (1895)
*Amanita* spp. (Amanitaceae)	Not stated	*Muchomor*	Gastrointestinal perforation	Digestive	Rostafiński (1883)
*Boletus* *edulis* (Boletaceae)	*Boletus* *edulis*	*Borowik*	“*Skuła*”	Skin	Wereńko (1896)
*Cetraria* *islandica* (Parmeliaceae)	*Lichen* *islandicus*; *Cetraria* *islandica*; *Lichen* *Island*; *L.* *Islandicus* L.; *Lichen* *Islandicus*	*Islandskiy* *mokh*, *płucnik*, *porost* *iślandski*, *pluśnik*, *mech* *szląski*, *zemlianaja* *ziabra*, *szlańskij* *moch*	Chest diseases	Respiratory	Friebe (1805); Ludwig (1905)
			Cough	Respiratory	Muszyński (1927); Bobrovskiy (1863); Friebe (1805)
			Kids diseases	General and Unspecified	Orzeszkowa (1891)
			Lung diseases	Respiratory	Ludwig (1905); Bermann, (1904)
			Tuberculosis	Respiratory	Muszyński (1927); Bobrovskiy (1863); Jundziłł, (1791); Wereńko (1896); Friebe (1805)
*Claviceps* *purpurea* (Clavicipitaceae)	*Secale* *cornutum*	Not stated	Weak stomach	Digestive	Friebe (1805)
			Oxytocic	Pregnancy, childbearing, family planning	Alksnis (1894)
*Clavulina* *coralloides* (Hydnaceae)	*Clavaria* *coralloides* L	*Kozia* *bródka*	Stomachache	Digestive	Kolbuszowski (1896)
*Clavulina* *coralloides* (Hydnaceae)	*Merisma* *fallax*	*płaskosz* *grzebieniasty*	Stomachache	Digestive	Kolbuszowski (1896)
*Craterellus* *undulates* (Hydnaceae)	*Helvell* *crispa*	*Mitra*	Evil eye	Culture-bound syndrome	Kolbuszowski (1896)
*Daedalea* *quercina* (Fomitopsidaceae)	*Daedalea* *quercina* Pers	*Skrypiel*	Mosquitoes	General and unspecified	Pietkiewicz (1928)
*Fomes* *fomentarius* (Polyporaceae)	*Polyporus* *fomentarius*, Fr	*Trutovik* *aptechnyy*, *ognivnyy*, *khirurgicheskíy*	Hemostatic	Blood	Cholovskiy (1882)
*Fomitopsis* *betulina* (Fomitopsidaceae)	*Birkenschwamm*	*Köbjas*	Diarrhoea	Digestive	Wiedemann (1876)
			Hamper diarrhoea	Digestive	Luce (1829
*Fomitopsis* *officinalis* (Fomitopsidaceae)	*Boletus* *laricis*	Not stated	Abdominalgia	Digestive	Alksnis (1894)
*Gymnosporangium* *clavariiforme* (Gymnosporangiaceae)	*Tremella* *juniperina*	Not stated	Chapped hands	Skin	Wiedemann (1876)
*Leccinum* *scabrum* (Boletaceae)	*Boletus* *scaber*, Bull	*Berezovik*, *chernyy* *grib*, *obabok*, *ababok*, *podobabka*, *grib*, *lepšė*	Cancer	General and Unspecified	Cholovskiy (1882)
			Rotten wounds	Skin	Cholovskiy (1882)
			Abscess	Skin	Petkevič, (1911)
*Lobaria* *pulmonaria* (Lobariaceae)	*Lobaria* *pulmonaria*, *L.*; *L.* *Pulmonarius* L*.*; *Lichen* *pulmonarius*	*Seinoles*; *kopsa* *rohhud*; *lischai*, *moch* *plijuschtschewoi* *(karastnaja—trawa)*; *porost* *płucni*k	Cauterisations	Skin	Alksnis (1894)
			Dissolving	General and Unspecified	Friebe (1805)
			Rhagades	Skin	Alksnis (1894)
			Sheep cough	Respiratory	Friebe (1805)
			Sore points	Skin	Alksnis (1894)
			Strengthening	General and Unspecified	Friebe (1805)
			Tuberculosis	General and Unspecified	Jundziłł, (1791)
*Lycoperdon* sp. (Agaricaceae)	*Lycoperdon*	*Purchawki*, *bzduchi*, *porchauka*, *purchawka*, *kukurbezdalis*, *dozhdevik*	Cuts	Skin	Petkevič, (1911)
			Hemostatic	Blood	Kolbuszowski (1896)
			Rotten wounds	Skin	Federowski (1897); Kolbuszowski (1896)
*Lycoperdon* *perlatum* (Lycoperdaceae)	*Lycoperdon* *Bovista*; *Lycoperdon* *Bovista* *L*	*Puhpedis*; not stated; *doschschewik*, *pruchauka*	Hemostatic	Blood	Friebe (1805); Hoelzl (1861)
			Abortifacient	Pregnancy, childbearing, family planning	Hoelzl (1861)
*Morchella* *esculenta* (Morchellaceae)	*Helvella* *esculenta*	Mitra	Evil eye	Culture-bound syndrome	Kolbuszowski (1896)
*Morchella* *sp.* *(Morchellaceae)*	*Morchella*	Not stated	Furunculosis	Skin	Alksnis (1894)
			Ulcers	Skin	Alksnis (1894)
*Phellinus* *igniarius* (Hymenochaetaceae)	*A.* *Igniarius* L.; *Boletus* *igniarius*, L	*Hubka*, *trutovik* *goryuchi*	Hemostatic	Blood	Jundziłł, (1791); Cholovskiy (1882)
*Phallus* *impudicus* (Phallaceae)	*Phallus* *impudicus*; *Phallus* *impudiens* Lin	*Jooksja* *rohhi*, *elli* *sibbul*, *sramotnik* *śmierdzący*, *vaniučy* *hryb*, *pampon*, *wiesiołek*	Flies	General and Unspecified	Kolbuszowski (1896)
			Genital ulcers	Male genital	Muszyński (1927)
			Gout	Endocrine/metabolic and nutritional	Luce (1829
			Rheumatism	Musculoskeletal	Luce (1829
*Saccharomyces* *cerevisiae* (Saccharomycetaceae)	*Hefe*	Not stated	“*Cast* *out* *bristles*” (most likely blackheads, dirt clods or raising epidermal cells; cleaning due to enhanced sweating)	Skin	Alksnis (1894)
			Diarrhoea	Digestive	Alksnis (1894)

### Diachronic comparison of fungi use

**Fig. 4:**
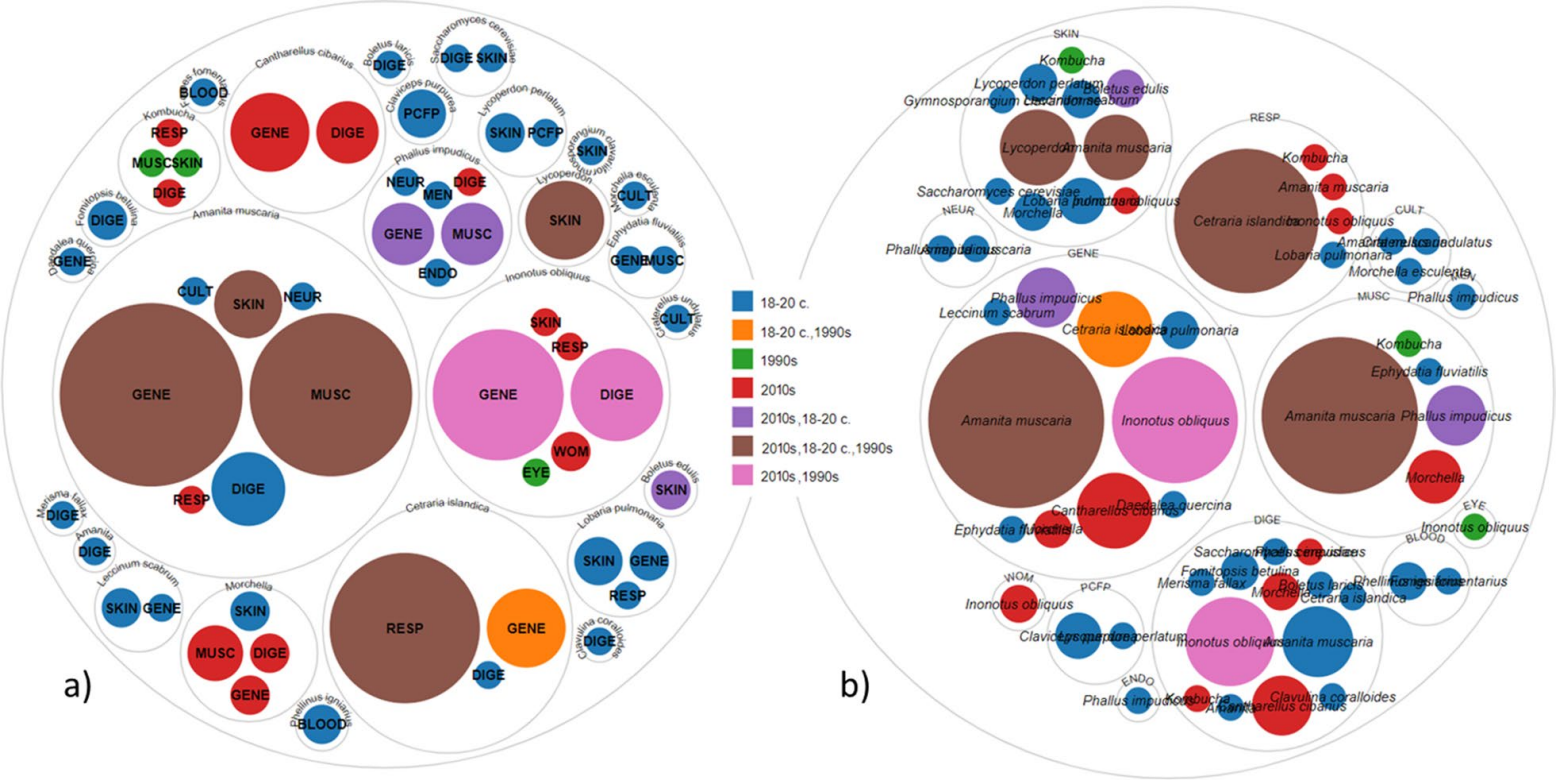
The diachronic distribution is arranged by uses **a** and taxa **b**

Only six of the 22 taxa historically used have been retained (Fig. 4a). The only taxon introduced recently was *Cantharellus* *cibarius*. The use of two folk taxa was acquired during Soviet times. For one of them, *Inonotus* *obliquus*, the circulation of various recent uses was observed, with uses in the digestive and general disease categories being of longer duration (used now and also mentioned with regard to past uses). The other is the emic taxon *Kombucha*, which was little used. The use of *Morchella* has completely changed, with the single traditional use having been discontinued and three new disease categories recorded during fieldwork.

There were several disease categories historically treated with fungi which have now been abandoned, the most notable being culture-bound syndromes, pregnancy-related (mainly abortive) uses and blood-related (coagulation) uses, while eye-related uses are of recent origin (Fig. 4b). One traditional use (*Cetraria* *islandica* for general diseases) was remembered as a past use (e.g., abandoned recently).

## DISCUSSION

Prior studies have underestimated or overlooked the importance of transmitting knowledge about the medicinal uses of fungi through written sources. The disparity between the shortlist of fungi and lichen currently used (nine) and the longer list from nineteenth-century written sources from the same region (22) signals significant erosion of the ethnomedicinal knowledge of fungi. Most of these recorded uses or specific applications were reported by a single person. This fragmentation and inconsistency indicates a lack of consensus within the communities regarding the medicinal use of fungi. Although there are still some traces of historical uses at the disease category level, the overlap for emic diseases is minimal.

Several factors have driven these changes. For example, the epidemiological situation in the region has evolved; however, when considering fungi uses, these changes are minimal. While diseases like malaria and culture-bound illnesses have faded, most of the uses were related to diseases that still exist and are treated with fungi. Living standards and access to affordable and effective medicines also impact fungi use.

**Fig. 5:**
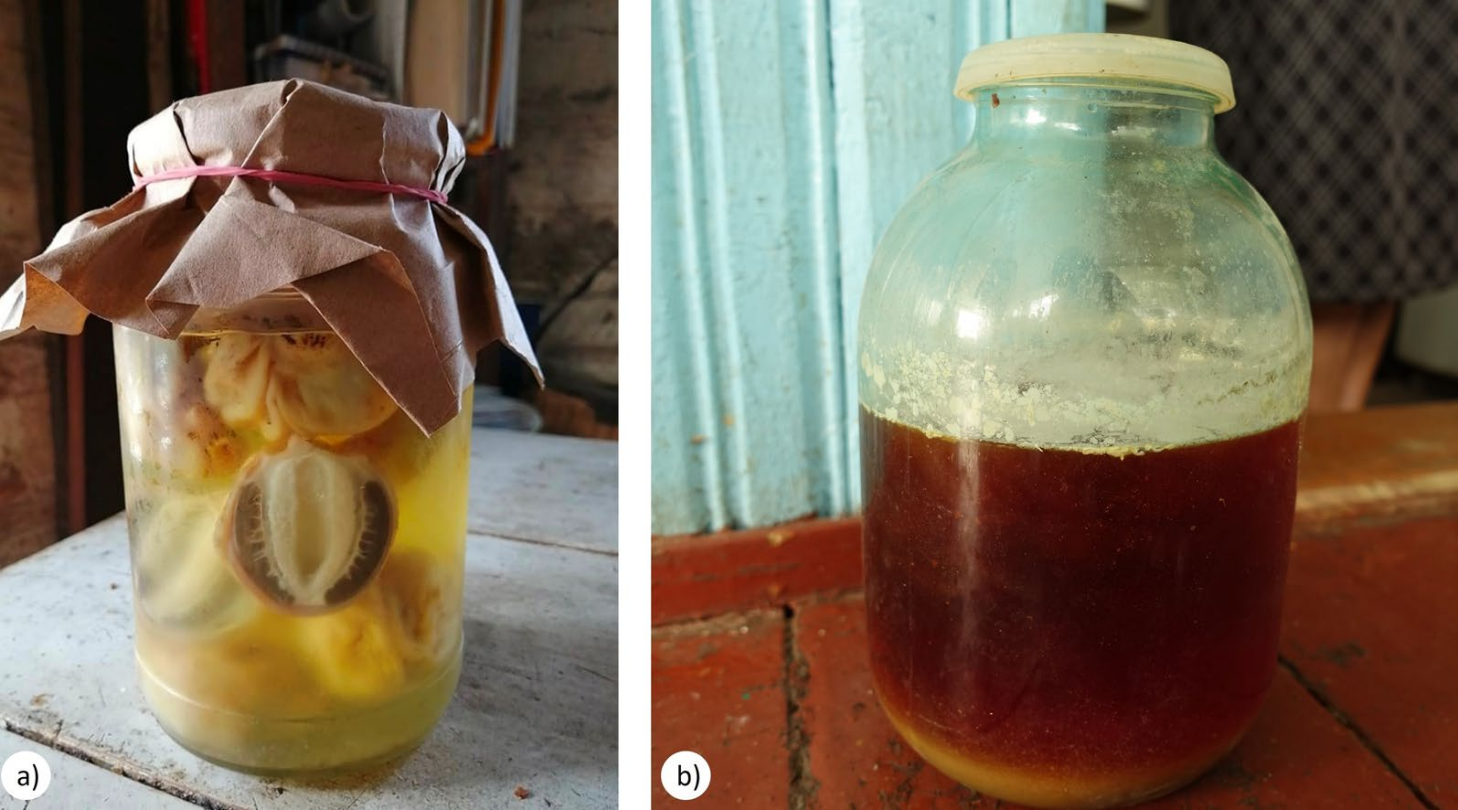
Alcoholic infusion with **a** *Phallus impudicus*, **b** *Amanita muscaria*, Belarus. Credit: JP, 2018–2019

Nevertheless, in relatively prosperous Finland, there have been numerous recent records of the use of *Inonotus* *obliquus*, possibly influenced by intensive promotion. In contrast, despite the widespread consumption of fungi for food in remote areas of Romania, none are currently used for healing (Stryamets et al. 2022). Meanwhile, in Poland, which is a post-socialist country (and formerly a Soviet satellite state), a few locals reported two traditional uses that align with those listed in books published from the late eighteenth century to the early twentieth century (*Amanita* *muscaria* against flies and *Boletus* for skin diseases). A higher diversity of taxa and uses was observed in Russia and the countries administered by the Soviet Union. The Polish and Lithuanian communities in Belarus have maintained a continuous tradition of using fungi for medicinal purposes, as illustrated in Fig. 5.

Our research findings indicate that the predominant source of information concerning the use of fungi for treatment is what can be termed “public knowledge”. This type of knowledge refers to a shared body of information within a community disseminated through social relations (Östling 2020). For example, among Polish and Lithuanian interviewees from the triangle between Poland, Belarus and Lithuania, the most common response was “*to* *jest* *od* *dawna* */* *tai* *yra* *nuo* *seno*” [approximately, “It has been around for ages”]. According to our interviewees, this knowledge was connected with evident and largely reliable information.

On the one hand, as with other types of ethnomedicinal knowledge, ethnomycological information passed down through family connections remains a reliable source for our interviewees (e.g., Mattalia et al. 2020). Indeed, a vertical mode of transmission was reported in all study cases except among Finns from Finland and Ukrainian Romanians. “*Kadais* *kai* *baisiai* *musės* *buvo* *tai* *musiomirus* *tep* *cukru* *užpildzinėjo*, *ar* *jos* *gaišo* *tos* *musės* *ar* *negaišo*, *ba* *jų* *buvo* *debesiai* *tų* *musių.* *An* *lango* *padėdavo* *tuos* *grybus* *musiomirus*, *cukru* *užpyldavo* *ir* *jau* *ci* *tos* *musės* *turės* *gaišt* (*…*) *Nu* [*tėvai* *darė*] *Da* *kap* *sako* *močiutės* *darė* *tep*”. [In the past, when there were scary loads of flies, they used to pour sugar over fly agarics to make those flies died [from this method] or not (…), but there were clouds of them. They placed those fungi, fly agarics, on window sills and poured sugar over them, expecting the flies to die. Yes, parents and, as they say, grandmothers did this] (Lithuanian, female, 66 years old, Poland). Across all study cases, many interviewees emphasised the significance of the successful or unsuccessful experiences of close relatives concerning the use of fungi, apart from the use of *Cantharellus* *cibarius* and *Boletus* *edulis*.

On the other hand, we observed intense knowledge circulation (Prakofjewa et al. 2023; Sõukand et al. 2024) through horizontal information exchange (Soldati et al. 2015). This means that the knowledge was shared among individuals within the local community rather than being passed down vertically through generations. It is crucial to note that this information was often questionable for interviewees and required confirmation from other sources. This was especially true for the use of *Amanita* *muscaria*, *Cantharellus* *cibarius*, *Cetraria* *islandica*, *Inonotus* *obliquus* and *Phallus* *impudicus*.

Regarding the use of written sources as an information reference (Prakofjewa et al. 2022), our interviewees from Finland, Belarus, Poland, Lithuania, Estonia and Russia emphasised the importance of books, local newspapers and the Internet in acquiring new knowledge about fungi usage. For instance, “*Vot* *ya* *delala* *nastoyku* *chagi*, *svekor* *kogda* *zabolel*, *pri* *onkologii.* *Nu* *vot* *opyat’* *zhe* *v* *etikh*, *v* *starom* *spravochnike* *lekarstvennykh* *trav*, *ya* *tam* *retsept* *nashla*” [I used to make chaga tincture for my father-in-law when he got sick, for oncology. And once again, I found the recipe in that one, in an old reference book of medicinal herbs] (Russian, female, 61 years old, Republic of Karelia, RF). Interviewees often mentioned using random herbals to confirm and complement existing public knowledge and family or community experiences. *Amanita* *muscaria*, *Cantharellus* *cibarius*, *Inonotus* *obliquus* and *Phallus* *impudicus* were mentioned in this regard. Books served as a valuable source for confirming information related to the specific usage of *Cetraria* *islandica*. On the other hand, the Internet provided new knowledge for Estonians, Karelians from Finland and Ukrainian Hutsuls about using *Inonotus* *obliquus* and *Amanita* *muscaria*. “*Kui* *mul* *oli* *eelmine* *aasta* *borrelioos* *siis* *määrati* *mulle* *antibiootikumi* *kuur* *kuid* *see* *ikka* *seda* *elukvaliteeti* *paremaks* *ei* *teinud.* *Ma* *mingi* *6–7* *kuud* *ikka* *olin* *selline* *et* *arvasin*, *et* *mind* *pikalt* *enam* *pole.* *Ja* *siis* *lugesin* *selle* *tšaga* *ehk* *kasekäsna*, *musta* *pässiku*, *kohta* *Internetist.* *Mul* *seal* *Otepääl* *tuttavad* *ütlesid*, *et* *Saaremaalt* *keegi* *mees* *seda* *korjab* *ja* *müüb*, *ja* *inimesed* *tellisid* *temalt*”. [When I had Lyme disease last year, I was prescribed antibiotics, but it still did not improve my quality of life. For about 6 or 7 months, I was still in a state where I thought I would be gone soon. And then I read about this chaga, or birch bark beetle, on the Internet. Some people I knew in Otepää said that a guy from Saaremaa was picking and selling it, and people were ordering from him] (Võro, female, 43 years old, Estonia). During the Soviet period, the established medical system was centralised by the state, where the government played a significant role in the delivery, financing and planning of healthcare services. This system was designed to provide universal health coverage to all citizens (Field 1957). The procurement practices, as a part of the centralised Soviet medical system, actively facilitated the continuous use of *Cetraria* *islandica* and *Inonotus* *obliquus* among Võro and Seto communities in Estonia. Notably, school procurement also played a vital role in the circulation of knowledge related to these medicinal resources. This implies that the institutional support provided by the Soviet medical system, coupled with educational initiatives like school procurement, significantly contributed to the sustained use and dissemination of traditional medicinal practices within the studied communities: “*Kasekäsni* *korjati* *ja* *osteti* *kokku*, *see* *on* *ka* *ravimtaim.* *Koolis* *saadeti* *metsa* *otsima.* *Veneajal* *oli*, *et* *niipalju* *peate* *andma.* *Mina* *proovisin* *ise* *ka* *seda* *vähi* *vastu.* *Teed* *juppideks* *ja* *teed* *kohviveskiga* *pudedaks* *ära* *ja* *siis* *keedan* *ära.* *Panen* *siis* *jahtuma* *ja* *joon.* *Ma* *päevas* *pooleliitrise* *purgikaupa* *seda* *joon.* *Mul* *on* *vähk*, *eesnäärme* *vähk.* *Kas* *aitab*, *ei* *tea* *aga* *räägitakse*, *keegi* *rääkis*, *et* *aitab*” [*Inonotus* *obliquus* was picked and bought together; it is also a medicinal plant. At school, we were sent to the forest to forage. In Russian times, there were norms on how much you had to collect. I tried it myself against cancer. You cut it into pieces, make it soft with a coffee grinder, and then boil it. Then I let it cool and drink it. I drink a half-litre can of it a day. I have cancer, prostate cancer. Whether it helps, I do not know, but they say somebody said it helps] (Seto, male, 57 years old, Estonia).

We generally found a notable positive attitude towards the medicinal applications of *Amanita* *muscaria*, *Inonotus* *obliquus*, *Cetraria* *islandica* and *Cantharellus* *cibarius*. The predominantly positive perception of the medicinal use of these fungi was linked to their beneficial effects within families and communities. However, a positive attitude was assured for specific fungi because of the dissemination of strong public knowledge. However, doubts and negative perceptions were explicitly recorded for *Inonotus* *obliquus* and *Cetraria* *islandica*. The negative attitudes towards *Cetraria* *islandica* mainly stem from its distinctive taste. People often acknowledged its potential health benefits but noted its unpleasant taste: *“Põdrasammal* *ehk* *islandi* *sammal* *selle* *teed* *olen* *ka* *joonud* *on* *köha* *vastu.* *Kes* *seda* *aga* *kannatas* *juua* *see* *on* *ju* *hullult* *mõru.* *Olen* *oma* *lastelegi* *teinud.* *Mäletan* *et* *mingil* *ajal* *oli* *mul* *väga* *suur* *pinge* *ja* *köhisin* *vist* *kuu* *aega* *järjest* *ja* *siis* *ma* *keetsin* *teda*, *ta* *muutus* *kohe* *süldiks.* *Siis* *ma* *sundisin* *ennast* *seda* *jooma”* [I have drunk this tea too. It is good for coughs. But for whoever drinks it, it is insanely bitter. I have made it for my children. I remember at one time, I was very tense and coughed for probably a month straight, and then I boiled it, and it became like pudding. Then I forced myself to drink it] (Seto, female, 61 years old, Estonia). Despite its perceived goodness for health, the taste acts as a deterrent. In the case of *Inonotus* *obliquus*, negative or doubtful sentiments primarily arise from the lack of necessary knowledge about the specificity of its use (e.g., exact dosages). People expressed uncertainty about its effectiveness, as it seems to yield inconsistent results, sometimes beneficial and other times not. The lack of clarity on proper dosing contributes to this hesitancy: *“Nu* *i*, *konechno*, *ochen’* *[rasprostranena]* *chaga.* *No* *yeye* *nado* *znat’*, *kak* *pravil’no* *vymachivat’*, *natirat’* *na* *terke* *nado*, *potom* *zavarivat’.* *Ona* *voobshche* *dazhe* *protiv* *onkologicheskikh* *zabolevaniy”* [And, of course, very [widely spread] chaga. But you need to know how to soak it properly, grate it, then brew it. It is generally even effective against cancer] (Belarusian, female, 68 years old, Republic of Karelia, RF).

**Fig. 6:**
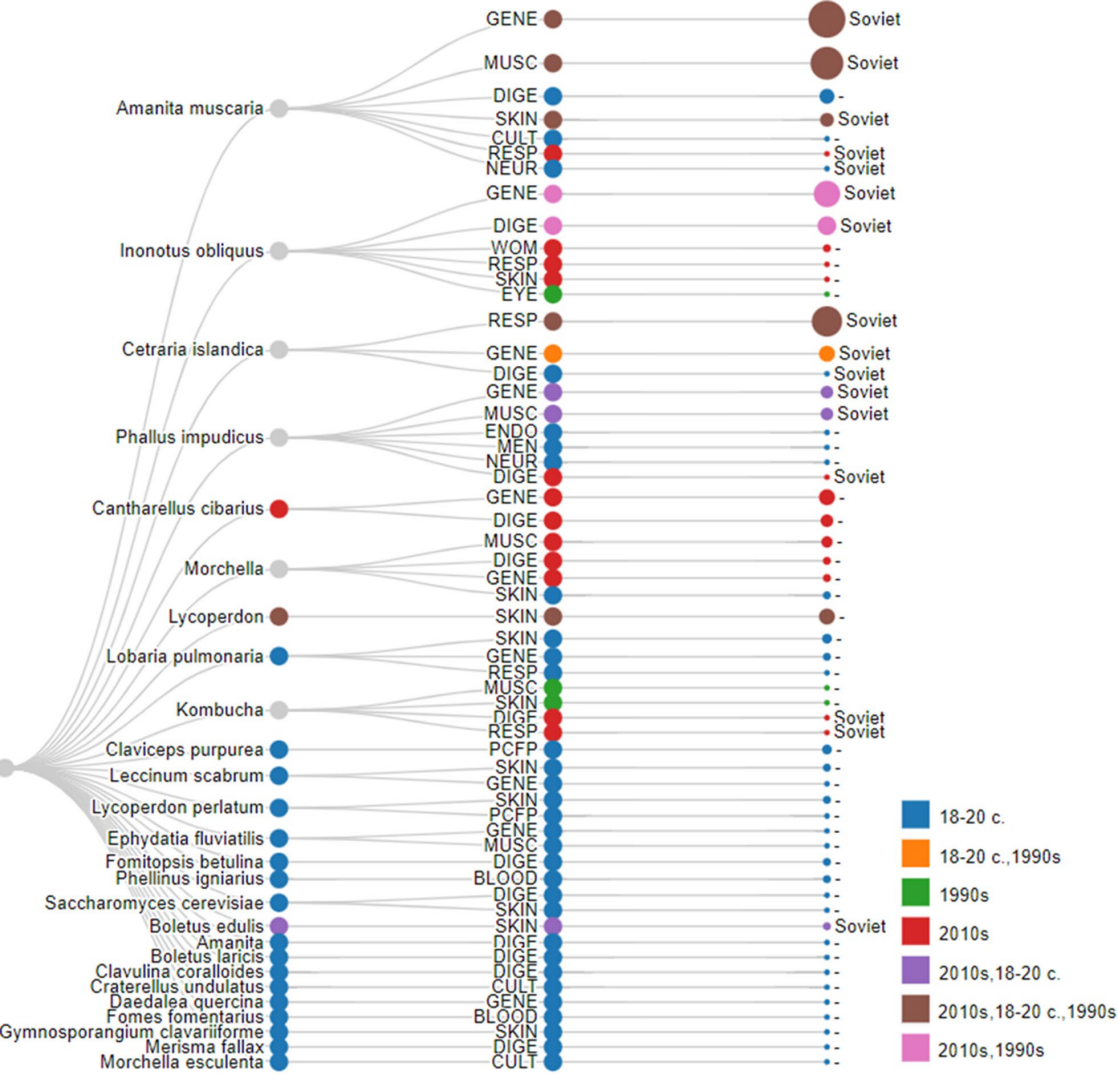
Correlation between promotion in Soviet books and documented time of use

Comparing our field results with herbals popularising the medicinal use of fungi published during the time of the Soviet Union, we can clearly observe that the use of specific fungal species could continue if the taxon (and its specific use) was mentioned in the centralised medicine of countries affected by centralisation (Fig. 6). None of the fungal species absent from the written sources continued to be used for medicinal purposes. We recorded specific explanations regarding the uses of *Cantharellus* *cibarius* that suggest a notable impact of modern written sources and the Internet on the circulation of traditional ecological knowledge.

The diverse applications of *Inonotus obliquus* could be linked to ongoing experimentation and increasing prominence in popular literature within studied regions. Furthermore, we identified two specific uses endorsed by Soviet herbals that did not endure over time, likely owing to their highly specialised manner of application.

Nowadays, the European Food Safety Authority (EFSA) and the European Medicines Agency (EMA) are two key agencies involved in the regulation and approval of the use of medicinal fungi in the European Union. The EU Directive 2002/46/EC generally harmonised the legislation relating to food supplements containing vitamins and minerals. Although not directly mentioned, medicinal fungal species are not categorised as such due to their nutritional benefits when they are marketed. The regulatory environment for use of medicinal fungi in Russia, Belarus and Ukraine is different in each country. In Russia, the regulation of medicinal mushrooms falls under the broader umbrella of dietary supplements and traditional medicine of the Federal Service for Surveillance in Healthcare. Belarus has a regulatory framework that is somewhat similar to Russia. Products like medicinal mushrooms intended for health purposes must undergo state assessment of quality, safety and efficacy. This process is less rigorous for dietary supplements compared to pharmaceuticals. Ukraine’s approach to regulating medicinal products, including those derived from mushrooms, is in transition, moving towards European standards. All three countries require that products be registered and proven safe for consumption. However, despite the most recent legislation, most interviewees sourced their medicinal fungi directly from the forest, apart from *Cetraria* *islandica* and *Inonotus obliquus* which are available on both sides of the former border as popular pharmaceutical products in drugstores.

## CONCLUSIONS

The consistent use of fungi for medicinal purposes across the western borderlands of the former Soviet Union has shown a strong correlation with public awareness and positive family experiences in healing with fungi. All current uses of fungi were either supported by or directly inspired by published sources such as books, local newspapers and the Internet. Notably, the analysis of official Soviet herbals revealed that the taxa (and their respective uses) endorsed and supported by the centralised Soviet system were frequently reported by the studied ethnic groups. These Soviet herbals conveyed official medical knowledge propagated by local doctors, medical assistants and pharmacists within the procurement system.

A significant portion of the historical written sources did not report the exact applications of fungi. During the current fieldwork, we observed a notable decline in the diversity of medicinal use and fragmentation of that knowledge. Crucially, taxa or uses not included in the official discourse ceased to circulate. Therefore, we advocate for a more in-depth exploration of the influence of literature and general medicinal discourse on ethnomedicine and the utilisation of fungi.

The current research has highlighted that recognising and acknowledging the knowledge passed down through generations can empower local communities, preserve natural and cultural heritage, and underscore the potential use of natural resources for medicine. Overall, this study strengthens the idea that coloniality, characterised by knowledge standardisation and centralisation, among other factors, affects the documentation of local uses included in historical written sources. LEK can ultimately survive if its place-based nature (what we term “situativity”) is intentionally fostered across various knowledge systems. Acknowledging this can pave the way for a holistic approach to healthcare and promote an inclusive and more respectful exchange of knowledge between disparate medical systems.

### Supplementary Information

Appendix A1 – provides the tabulated field results and ethnographic data from the books.

Appendix A2 – provides the crude data reflecting the correlation between the circulation of taxa use and their promotion in Soviet popular herbals.

## Data Availability

The datasets used and analysed during the current study are available from the corresponding author upon reasonable request.

## Abbreviations

DNA: Deoxyribonucleic acid
EU: European Union
ITS: Internal transcribed spacer
LEK: Local ecological knowledge
PCR: Polymerase chain reaction
RF: Russian federation
TEK: Traditional ecological knowledge
UR: Use report
USSR: Union of Soviet Socialist Republics
